# Physical education environment and student physical activity levels in low-income communities

**DOI:** 10.1186/s12889-020-8278-8

**Published:** 2020-01-31

**Authors:** Soyang Kwon, Sarah Welch, Maryann Mason

**Affiliations:** Ann & Robert H. Lurie Children’s Hospital of Chicago Stanley Manne Children’s Research Institute, 225 E Chicago Ave. Box 157, Chicago, IL 60611 USA

**Keywords:** Elementary school, Middle school, High school, Lesson context, Motor content, Teacher behavior, SOFIT

## Abstract

**Background:**

The purpose of this study was to examine the association of physical education (PE) class characteristics, such as lesson context, teacher’s physical activity (PA) promotion behavior, and lesson location, with student engagement in moderate- and vigorous-intensity physical activity (MVPA) during PE lessons in elementary school (ES), middle school (MS), and high school (HS).

**Methods:**

The study sample included 2106 PE classes from 40 schools located in low-income communities. The System for Observing Fitness Instruction Time (SOFIT) was used to assess lesson context, teacher’s behavior, and student PA during PE lessons. Mixed models were used to examine the association between PE class characteristics and the probability of meeting the recommended level of MVPA during PE lessons (MVPA ≥50%), accounting for within-school random effects and school characteristics.

**Results:**

Almost all PE classes (90%) with ≥60–70% of lesson time spent in motor content and ≥ 10–20% in teacher’s in-class PA promotion met the recommended level of MVPA across the school levels. More specifically, among the sub-categories of motor content, more lesson time spent in fitness was significantly associated with MVPA ≥50% in all school levels. However, more lesson time spent in game play was a significant factor only in ES (OR = 2.1; 95% CI = 1.4–3.0). Outdoor lessons were a significant factor in ES (OR = 5.3; 95% CI = 3.1–9.0) and MS (OR = 21.0; 95% CI = 6.3–69.4), but not HS (OR = 1.4; 95% CI = 0.6, 3.2).

**Conclusions:**

PE lessons with higher motor content and higher teacher’s in-class PA promotion are more likely to meet the recommended level of MVPA in all school levels. However, the sub-categories of motor content and lesson location could impact student MVPA differently by school levels.

## Background

Physical activity (PA) has numerous health benefits for children and adolescents [1]. Schools are a valuable setting to provide children with PA opportunities [2]. In particular, physical education (PE) is a direct opportunity for PA in school. The US Centers for Disease Control and Prevention [3] and UK Associations for Physical Education [4] advises that students should engage in moderate- and vigorous-intensity physical activity (MVPA) for at least 50% of PE lesson time, to gain appropriate health and academic benefits. Recent meta-analyses on MVPA during PE lessons in primary schools (elementary schools) [5] and secondary schools (middle and high schools) [6] by Hollis and colleagues suggest that, based on observation measures such as the System for Observing Fitness Instruction Time (SOFIT), percent time spent in MVPA was 58% for elementary PE classes and 44% for secondary school PE classes. Especially in secondary schools located in low-income communities, MVPA during PE was reported to be low (39%) [7], which is aligned with the finding that children and adolescents with economically disadvantaged backgrounds are less active [8, 9]. Fifth graders in Vietnam were reported to engage in MVPA for 33% of PE class time [10]. The meta-analysis for elementary schools [5] suggests that, on average, elementary school PE classes meet the recommended level of MVPA. However, the meta-analysis included very few studies (*n* = 4). In contrast to the reviews by Hollis et al. [5, 6], another review [11] reported that MVPA levels during PE lessons were higher in secondary schools than elementary schools. A few studies that were not included in the two reviews [5, 11] also reported a lower MVPA level in elementary schools than the meta-analysis: 42% in UK elementary schools [12] and 38% in US elementary schools [13]. These findings indicate that the majority of schools in all levels (elementary, middle, and high school) may not meet the recommended MVPA levels, especially those located in low-income communities.

To increase MVPA during PE lessons, it is critical to understand the factors that facilitate MVPA during PE lessons. In particular, modifiable class-level factors, such as lesson context and instructor behaviors, will have impacts to increase overall MVPA at a class level. Zhou and Wang [14] reviewed correlates of MVPA in secondary school PE and concluded that at the class level, class gender (males-only), lesson context (team games or less time spent on knowledge content), and lesson location (outdoor) were consistently reported to be associated with MVPA. In an elementary school study, Skala et al. [13] reported positive associations between outdoor PE classes, active lesson context, smaller class size, and shorter class length and MVPA. As students are in various developmental and learning stages across school levels, their PA behaviors could be affected by different factors. Identifying specific target factors by school level can aid in designing effective and tailored interventions and policies. However, few studies have ascertained the different environmental factors that influence MVPA during PE by school level. The purpose of this study was to examine the association of lesson location, lesson context, teacher’s PA promotion behavior, class size, lesson length, and grade with student MVPA during PE lessons in elementary, middle, and high schools.

## Methods

### Participants

This study used data collected for a single-county evaluation project in Illinois, U.S.A. that evaluated student PA levels during PE classes in public schools. The geographic area served by this evaluation is considered to be a suburban area with approximately 2.5 million people (57% non-Hispanic white, 19% Hispanic, 16% black, and 16% Asian). Most public schools in this suburban area were organized into elementary, middle, and high schools. The state of Illinois had a daily PE requirement, although waivers and exemptions were allowed. The state of Illinois had no mandatory policy, but provided recommendations, regarding a PE specialist and curriculum. The evaluation focused on nine school districts in the county with ≥40% of students eligible for free- or reduced-price lunch. All nine districts were invited to and agreed to participate in the evaluation. The evaluation involved six waves of SOFIT assessments over two school years (fall 2015, early winter 2016, late winter 2016, spring 2016, fall 2016, and spring 2017). Among the total of 43 schools within the nine districts, three schools (7%) were disengaged from the evaluation during the scheduling period for the first or second assessment. Of the remaining 40 schools, 26 were elementary schools, six were middle schools, and eight were high schools. After the first assessment in fall 2015, schools were offered various supports, such as PE teacher training, school administrator training for PA and PE policies, and technical assistance for policy improvement. Trainings were designed to provide the information needed for school staff to make improvements to their PE policies, as well as capacity building skills and strategies for addressing barriers in the improvement process. Although PE teacher’s participation in the PE training was voluntary, the majority of PE teachers participated in the training. As we found that the wave 1 data had a large proportion of missing data or outliers (> 10%) and student PA levels estimated in wave 1 were substantially different from those in waves 2 to 6, we excluded the wave 1 data and used the waves 2 to 6 data for this report.

At each wave, two typical school days per school were selected to conduct the SOFIT observations. All PE classes scheduled on those 2 days were observed. The number of PE classes per day varied, ranging from two to eight. A total of 2106 PE classes across the 40 schools were observed. For the current analysis, we excluded six PE classes that were solely for students with special needs, 24 PE classes that were for preschoolers, and 14 PE classes that were observed for less than 16 min, resulting in 2063 PE classes.

### Measurements

The System for Observing Fitness Instruction Time (SOFIT) was used to assess student PA, lesson context, and teacher behavior. The SOFIT is a direct observation method that utilizes a momentary time sampling and interval recording system to assess PA levels, lesson context, and teacher behavior over a designated class period [15]. SOFIT evaluates student activity level in five categories: lying down, sitting, standing, walking and vigorous activity. Lesson context is categorized into general content (transition, management, and break), knowledge content (physical fitness, general knowledge, rules, strategy, social behavior, and technique), and motor content (fitness, skill practices, game play, and other). Teacher behavior is classified into three categories: when in-class PA or fitness is promoted (in-class PA promotion), when out-of-class PA or fitness is promoted (out-of-class PA promotion), and when neither in- nor out-of-class PA or fitness is promoted (no PA promotion). The definitions of all these categories can be found in the SOFIT manual [16]. The guidelines for SOFIT study reporting suggested by McKenzie and Smith [11] informed the data reporting of this report. The observer training process was conducted in accordance with the SOFIT manual [16]. Briefly, a project manager with extensive experience on SOFIT observations led the observer training. Observers completed classroom training, video analysis, and field practice. Classroom training included a general overview of the method, a detailed review of the protocol and question and answer sessions. The observers watched the SOFIT training and coding practice videos suggested in the manual. Finally, the observers observed a youth soccer practice. After the observation, they debriefed the experience. Discussion included a review of any data points for which codes between observers were discordant. The observers discussed and came to consensus on the appropriate code for that situation and notes were added to the internal protocol manual for consistent application of the code during data collection.

Five waves of SOFIT assessments were conducted in early winter 2016 (January and February), late winter 2016 (March to early April), spring 2016 (late April and May), fall 2016 (September and October), and spring 2017 (April and May). Research staff contacted a school representative and scheduled assessment visits (2 days per school at each wave). Only typical school days (e.g., no field day or no shorter day) were selected for the SOFIT assessment. Those 2 days were mostly two consecutive days. However, when scheduling for two consecutive days was not allowed, 2 days that were not on the same day were selected within 2 weeks (e.g., Monday and following week Tuesday). The research staff were given the PE class schedules for the scheduled assessment days. One observer conducted the SOFIT observation per PE class. At the onset of the PE class, four target students and one potential target student as a backup were selected to assess student PA. The observer observed student one for 4 min, then rotated the observation to the remaining three students for 4 min each until the lesson ended. The observer recorded student activity, lesson context, and teacher behavior every 20 s for the entire PE class, using a paper-form of the SOFIT coding sheet. The paper-form data were entered into an electronic database system that automatically summarized activity, lesson context, and teacher behavior per student and per class. For data quality control, data entry for 10% of the observed PE class sample was cross-checked by independent research staff.

In addition to the direct observation of student PA, the observer recorded descriptive characteristics of the PE class, such as grade level, lesson location (indoor, outdoor, or both), scheduled length of PE class, the number of PE teachers, and the number of students. All observed PE classes were gender-mixed classes, with approximately half males and half females. The exact class gender composition was not recorded. Data on school characteristics such as racial/ethnic composition of the student body and percentage of low-income students (determined by students who are eligible for free- or reduced-price lunch) were obtained from the www.illinoisreportcard.com website provided by the Illinois State Board of Education.

### Statistical analysis

To check for potential data entry errors, extensive descriptive statistics, including range, median, mean, and outlier tests, per class, per day, per school, and per wave were conducted. An activity level was evaluated as a percentage of PE class time (frequency) spent in the activity level. For example: If walking activity was recorded for 30 intervals during a 90-interval (that is, 30-min) observation, walking activity level was 33% (calculated as 30 divided by 90). Because the lying down activity was rare, we combined lying down and sitting. Walking and vigorous activities were also combined as MVPA. MVPA was then dichotomized as ≥50 and < 50%. Teacher behavior categories and lesson context categories were also evaluated as percentages in a similar manner. Lesson time spent in each of in-class PA promotion, motor content, game play content, and fitness content was dichotomized as higher and lower. The cut-points for dichotomization were determined based on the median value in PE classes with MVPA≥50%. Specifically, for in-class PA promotion, “higher” was defined as ≥20% for elementary school, ≥15% for middle school, and ≥ 10% for high school. For motor content, “higher” was defined as ≥60% for elementary school, ≥65% for middle school, and ≥ 70% for high school. For game play content, “higher” was defined as ≥20% for elementary and middle schools and ≥ 30% for high school. For fitness content, “higher” was defined as ≥30% for all school levels.

To calculate the number of students per PE teacher (student-teacher ratio), the total number of students was divided by the number of teachers in class. For the lesson location variable, “outdoor” and “both” categories were combined as “outdoor,” because only < 5% of the PE class sample occurred in “both” and MVPA levels for “outdoor” and “both” were similar. To assign a single numeric grade value for grade-mixed classes, a median value was used. For example, the grade value for a first and second grade-mixed PE class was 1.5. The grade value for Kindergarteners was assigned as 0. The number of male students observed (1, 2, 3, or 4) was calculated. School race/ethnicity was assigned based on the majority race/ethnicity of the student body (black, Hispanic, white, or other).

All statistical analyses were conducted separately by school level: elementary, middle, and high school. Means and 95% confidence intervals (CIs) of the main study variables were calculated. To examine the relationship of PE class characteristics and school characteristics with MVPA (≥50% or < 50%), chi-square tests and t-tests were conducted. To account for within-school random effects, mixed models were used to predict MVPA ≥50%. The first set of mixed logistic regression models (Model 1) included lesson location (outdoor vs. indoor), in-class PA promotion (high vs. low), and motor content (high vs. low) as the predictors of interest. To examine more detailed motor content of the PE lessons, the second set of mixed models (Model 2) included lesson location, in-class PA promotion, fitness content (high vs. low), and game play (high vs. low) as the predictors of interest. All mixed models were adjusted for majority race/ethnicity of the student body (black, Hispanic, vs. other) and percentage of low-income students (< 80% vs. ≥80%), because our prior study in the same geographic area showed that student MVPA was lower in minority-majority schools and lower income schools [9]. For middle schools only, the models were additionally adjusted for grade (8th vs. 6th or 7th), because bivariate analysis found that grade was a significant factor only in middle schools. For high schools only, the models were additionally adjusted for the number of male students observed (3 or 4 vs. 1 or 2), because bivariate analysis found that the number of male students observed was a significant factor only in middle schools. Based on the mixed models, odds ratios (ORs) and 95% CI were estimated. A significance level was set at 0.05. All analyses were performed using SAS 9.4 (Cary, NC).

## Results

Of the 40 participating schools, 19 were black-majority schools (> 50% black students), 13 were Hispanic-majority schools, and one was a non-Hispanic white-majority school. The remaining seven schools had no single race/ethnicity group that comprised > 50% of students. The median percentage of low-income students was 80%, ranging from 40 to 98%. Of the 40 participating schools, 36 schools completed all five assessments and the remaining four schools completed four assessments. In those four schools, one missed assessment was due to a schedule conflict.

Table 1 shows the characteristics of the PE classes. More detailed distribution statistics are provided in Additional file 1 Table S1. As expected, the number of students per teacher was significantly higher and class length was significantly longer in middle and high schools, compared to elementary schools. Teacher’s in-class PA promotion was significantly higher in elementary schools than middle or high schools. Elementary schools presented the highest time spent in inactive content (i.e., general and knowledge content), followed by middle schools, and high schools. Average PE lesson time spent in MVPA was 47% in elementary schools, 43% in middle schools, and 54% in high schools.

**Table 1:** Descriptive Characteristics of the PE class sample

	Elementary school (*n* = 1386)	Middle school (*n* = 255)	High school (*n* = 422)
	*n* (%)	*n* (%)	*n* (%)
Outdoor lesson	165 (12)	48 (19)	37 (9)
	Mean (95% CI)	Mean (95% CI)	Mean (95% CI)
Class size (#students), *n*	22 (22, 23)	46 (43, 49)	36 (34, 38)
Students per PE teacher, *n*	20 (20, 21)	33 (31, 35)	31 (30, 33)
Scheduled length of PE class, minutes	32 (31, 32)	46 (44, 48)	46 (45, 48)
Actual length of PE class, minutes	31 (31, 31)	41 (39, 43)	41 (40, 42)
Observation length of PE class, minutes	31 (31, 31)	41 (39, 43)	41 (39, 42)
Teacher behavior			
In-class PA promotion, %	19 (18, 20)	13 (11, 15)	9 (8, 11)
Out-of-class PA promotion, %	0.2 (0.2, 0.3)	0.3 (0.2, 0.4)	0.1 (0.0, 0.1)
No PA promotion, %	80 (79, 81)	86 (84, 89)	91 (89, 92)
Lesson context			
General, %	30 (30, 31)	32 (30, 34)	24 (23, 25)
Knowledge, %	14 (14, 15)	6 (5, 7)	3 (2, 3)
Motor, %	55 (55, 56)	62 (60, 64)	73 (72, 74)
Fitness, %	25 (24, 26)	22 (20, 25)	29 (26, 31)
Skill practice, %	10 (9, 11)	9 (7, 11)	3 (2, 5)
Game play, %	15 (14, 16)	27 (24, 31)	30 (27, 33)
Other, %	5 (4, 6)	3 (2, 5)	11 (9, 13)
Activity level			
Lying down/sitting, %	27 (26, 28)	30 (28, 33)	18 (17, 20)
Standing, %	26 (25, 27)	26 (24, 29)	28 (26, 29)
Walking, %	30 (30, 31)	33 (31, 35)	41 (40, 43)
Vigorous, %	16 (16, 17)	11 (9, 12)	13 (12, 14)
MVPA, %	47 (46, 47)	44 (42, 46)	54 (52, 56)

*CI* confidence interval; *MVPA* moderate- and vigorous-intensity physical activity; *PA* physical activity; *PE* physical education

Table 2 presents MVPA levels by class characteristics. Higher in-class PA promotion, higher fitness content, and lower general content were significantly associated with MVPA ≥50% across school levels. However, the association between game play content and MVPA was positive in elementary schools, while it was negative in middle schools. Overall, PE classes with higher motor content and higher teacher’s in-class PA promotion had 90% probability of MVPA ≥50%.

**Table 2:** Comparison of PE class characteristics between PE classes with MVPA< 50% and MVPA≥50%

	Elementary school			Middle school			High school		
	MVPA < 50%	MVPA ≥ 50%		MVPA < 50%	MVPA ≥ 50%		MVPA < 50%	MVPA ≥ 50%	
	*n* (%)	*n* (%)	*p*	n (%)	*n* (%)	*p*	*n* (%)	*n* (%)	*p*
PE class	792 (57)	594 (43)		165 (65)	90 (35)		166 (39)	256 (61)	
Grade^a^			0.12			0.06			NA
Lowest	222 (54)	190 (46)		47 (72)	18 (28)		NA	NA	
Middle	318 (60)	208 (40)		75 (68)	36 (32)		NA	NA	
Highest	252 (56)	196 (44)		43 (54)	36 (46)		NA	NA	
Lesson location			< 0.01			< 0.01			0.88
Outdoor	42 (25)	123 (75)		18 (38)	30 (62)		15 (41)	22 (59)	
Indoor	750 (61)	471 (39)		147 (71)	60 (29)		151 (39)	234 (61)	
	M ± SD	M ± SD	p	M ± SD	M ± SD	p	M ± SD	M ± SD	p
Male students observed, n	2.0 ± 0.8	2.1 ± 0.8	0.23	2.2 ± 0.9	2.1 ± 0.9	0.58	2.1 ± 0.9	2.4 ± 0.9	< 0.05
Class size (# students), n	22 ± 8	22 ± 7	0.65	41 ± 25	55 ± 31	< 0.01	35 ± 15	37 ± 21	0.33
Students per teacher, n	20 ± 6	21 ± 6	0.11	32 ± 17	34 ± 23	0.45	32 ± 12	31 ± 15	0.24
Actual length of PE class, min	31 ± 5	32 ± 5	< 0.01	40 ± 14	42 ± 20	0.35	41 ± 11	41 ± 14	0.85
In-class PA promotion, %	16 ± 17	23 ± 22	< 0.01	6 ± 10	26 ± 22	< 0.01	4 ± 7	13 ± 15	< 0.01
General content, %	36 ± 15	23 ± 10	< 0.01	37 ± 16	23 ± 10	< 0.01	29 ± 15	21 ± 10	< 0.01
Knowledge content, %	17 ± 11	11 ± 9	< 0.01	5 ± 9	8 ± 7	< 0.01	3 ± 6	3 ± 4	0.34
Motor content, %	48 ± 16	66 ± 14	< 0.01	58 ± 17	69 ± 11	< 0.01	69 ± 17	76 ± 12	< 0.01
Fitness, %	19 ± 19	33 ± 21	< 0.01	15 ± 12	36 ± 22	< 0.01	24 ± 24	32 ± 28	< 0.01
Skill practice, %	12 ± 17	9 ± 15	< 0.01	7 ± 16	12 ± 20	< 0.05	5 ± 14	2 ± 11	0.07
Game play, %	12 ± 17	18 ± 18	< 0.01	34 ± 28	16 ± 20	< 0.01	29 ± 29	31 ± 30	0.53
Other, %	5 ± 15	5 ± 17	0.49	3 ± 11	4 ± 16	0.41	10 ± 7	11 ± 8	0.62

*M ± SD* mean ± standard deviation; *MVPA* moderate- and vigorous-intensity physical activity; *NA* not applicable; *PA* physical activity; *PE* physical education

^a^For elementary school, the lowest was grades K-1, the middle was grades 2–3 and the highest was ≥grades 4–5. For middle schools, the lowest was grade 6, the middle was grade 7, and the highest was grade 8. Because most high school PE lessons were grade-mixed, MVPA levels by grades was not analyzed for high schools

As shown in Table 3, a lower percentage of low-income students was significantly associated with MVPA ≥50% in elementary and high schools. Some differences in MVPA levels were observed across the school race/ethnicity categories.

**Table 3:** MVPA levels by school characteristics and assessment times

	Elementary school			Middle school			High school		
	MVPA < 50%	MVPA ≥ 50%		MVPA < 50%	MVPA ≥ 50%		MVPA < 50%	MVPA ≥ 50%	
	*n* (%)	*n* (%)	*p*	*n* (%)	*n* (%)	*p*	*n* (%)	*n* (%)	*p*
Race/ethnicity			< 0.01			< 0.01			< 0.05
Black	484 (86)	78 (14)		112 (86)	19 (14)		59 (33)	120 (67)	
Hispanic	109 (22)	380 (78)		35 (47)	39 (53)		93 (46)	110 (54)	
Other^a^	199 (59)	136 (41)		18 (36)	32 (64)		14 (35)	26 (65)	
Disadvantaged students %			< 0.01			0.85			< 0.01
< 80%	416 (48)	446 (52)		86 (64)	48 (36)		26 (27)	72 (73)	
≥80%	376 (72)	148 (28)		79 (65)	42 (35)		140 (43)	184 (57)	
Season			0.60			0.79			0.18
Spring/fall	469 (57)	360 (43)		100 (65)	53 (35)		108 (42)	150 (58)	
Winter	323 (58)	234 (42)		65 (64)	37 (36)		58 (35)	106 (65)	
School year			0.81			0.09			0.76
2015–2016	459 (57)	348 (43)		92 (61)	60 (39)		101 (40)	152 (60)	
2016–2017	333 (58)	246 (42)		73 (71)	30 (29)		65 (38)	104 (62)	

*MVPA* moderate- and vigorous-intensity physical activity

^a^Other schools are racially/ethnically mixed (no majority-race/ethnicity) schools for elementary and middle schools and a white-majority school for high school

The mixed models showed that PE lessons with higher teacher’s in-class PA promotion (ORs ranged 5.3 to 7.6; *p* < 0.05) and with higher motor content (ORs ranged 3.0 to 5.7; p < 0.05) were more likely to meet the recommended level of MVPA in all school levels (Table 4). More specifically, among the sub-categories of motor content, fitness content was associated with MVPA ≥50% in all school levels (ORs ranged 1.4 to 11.5; p < 0.05). Higher game play content was positively associated with MVPA ≥50% only in elementary schools (OR = 2.1; 95% CI = 1.4, 3.0). An outdoor PE lesson was significantly more likely than an indoor lesson to meet the recommended level of MVPA in elementary and middle schools, but not in high schools.

**Table 4:** Mixed logistic regression models for PE classes with MVPA ≥50%

	Elementary school	Middle school	High school
	OR (95% CI)	OR (95% CI)	OR (95% CI)
Model 1			
Lesson location: outdoor vs. indoor	5.3 (3.1, 9.0)	21.0 (6.3, 69.4)	1.4 (0.6, 3.2)
In-class PA promotion: higher vs. lower^a^	5.3 (3.3, 8.6)	6.0 (2.0, 18.0)	7.6 (3.8, 15.3)
Motor content: higher vs. lower^b^	5.2 (3.4, 7.9)	5.7 (2.3, 14.1)	3.0 (1.9, 4.8)
Grade: 8th vs. 6th or 7th	NA^d^	2.2 (1.0, 4.6)	NA^e^
The number of boys observed: 3 or 4 vs. 1 or 2	NA^f^	NA^f^	1.6 (1.0, 2.5)
Model 2			
Lesson location: outdoor vs. indoor	6.0 (3.6, 10.2)	44.1 (12.1, 160.4)	0.9 (0.4, 2.1)
In-class PA promotion: higher vs. lower^a^	4.3 (2.7, 6.8)	4.5 (1.5, 13.9)	6.0 (3.0, 11.9)
Fitness content: ≥30% vs. < 30%	1.4 (1.0, 2.1)	11.5 (4.0, 33.4)	1.9 (1.1, 3.4)
Game play content: higher vs. lower^c^	2.1 (1.4, 3.0)	0.8 (0.3, 1.8)	1.1 (0.7, 1.8)
Grade: 8th vs. 6th or 7th	NA^d^	2.3 (1.1, 5.0)	NA^e^
The number of male students observed: 3 or 4 vs. 1 or 2	NA^f^	NA^f^	1.8 (1.1, 2.8)

*CI* confidence interval; *MVPA* moderate- and vigorous-intensity physical activity; *PA* physical activity; *PE* physical education

All mixed models were adjusted for school race/ethnicity and the proportion of disadvantaged students. The mixed models for high school were additionally adjusted for proportion of males observed

^a^“Higher” was defined as ≥20% for elementary school, ≥15% for middle school, and ≥ 10% for higher school. “Lower” was defined as < 20% for elementary school, < 15% for middle school, and < 10% for high school

^b^“Higher” was defined as ≥60% for elementary school, ≥65% for middle school, and ≥ 70% for high school. “Lower” was defined as < 60% for elementary school, < 65% for middle school, and < 70% for high school

^c^“Higher” was defined as ≥20% for elementary and middle schools and ≥ 30% for high school. “Lower” was defined as < 20% for elementary and middle schools and < 30% for high school

^d^Grade was not included in the mixed models for elementary schools, because grade was not associated with MVPA≥50% in a bivariate analysis shown in Table 2

^e^Grade was not included in the mixed models for high school, because most high school PE lessons were grade-mixed

^f^The number of male students observed was not included in the mixed models for elementary and middles schools, because it was not associated with MVPA≥50% in a bivariate analysis shown in Table 2

## Discussion

This study aimed to identify PE class characteristics that are associated with student MVPA. We examined these characteristics separately by school level. Our study found that higher lesson times spent in teacher’s in-class PA promotion and motor content were two universal factors that were associated with MVPA ≥50% across all school levels. In particular, outdoor class location showed the strongest association in middle schools, while in-class PA promotion showed the strongest association in high schools. Almost all PE classes (90%) across the school levels that spent ≥60–70% of lesson time in motor content and ≥ 10–20% of lesson time in teacher’s in-class PA promotion met the recommended level of MVPA. On the other hand, some factors were significant only for certain school levels. Outdoor lessons were associated with MVPA ≥50% in elementary and middle schools, but not in high schools. Additionally, higher game play content was associated with MVPA ≥50% only in elementary schools. This study was unable to detect the effect of lesson length within a school level, mainly because of a lack of variation in the lesson length.

To address rising inactivity [17] and obesity [18] problems among children and adolescents, school-based PE is identified as a target intervention opportunity to increase PA at a population level [19, 20]. Enhanced school-based PE is one of the 17 objectives in the Healthy People 2020 Physical Activity area [21]. School/education policies, for example, the Every Student Succeeds Act (ESSA), promote school-based PE by expanding the federal definition of a well-rounded education to include PE [22]. Most U.S. states mandate that a significant amount of school time be devoted to PE; 150 min per week for elementary schools and 225 min per week for secondary schools [19]. However, offering PE opportunities alone would not be sufficient, if students engage in PA only for a short amount of time during PE class. In fact, studies in the U.S. have reported that students on average engage in MVPA less than 50% of the PE lesson time [11]. This implies that the majority of PE classes would not meet the MVPA recommendation (MVPA≥50%) [3]. Furthermore, our prior study reported that students in low-income, majority-minority communities engaged in even lower PA at school [9]. This current study found that among schools in low-income communities, the percentage of PE classes that met the recommendation was 43% for elementary schools, 35% for middle schools, and 61% for high schools. The average time spent in MVPA during PE lessons was 47% for elementary schools, 44% for middle schools, and 54% for high schools. Assuming actual PE lesson length was 30 min for elementary schools and 40 min for middle and high schools, these results translate into 14 min of MVPA for elementary schools, 14 min for middle schools, and 24 min for high schools.

These indicate that school-based PE contributes only 23 and 40% to meeting a daily MVPA recommendation (60 min/day) [1] in elementary/middle schoolers and high schoolers, respectively. Unlike after-school programs or extramural sports programs that individuals or communities have different levels of access to, school-based PE can impact almost all children. Therefore, evidence-based interventions to increase MVPA during PE will help to increase the prevalence of meeting the daily MVPA recommendation.

To support the evidence-based interventions, the current study identified the PE class characteristics that were associated with MVPA by school level. We found that motor content and teacher’s in-class PA promotion were two universal factors that were associated with MVPA ≥50% across all school levels. However, beyond the associations, it is also important to recognize the differences in PE class context and teacher’s in-class PA promotion levels across school levels. It may be age-appropriate that elementary PE classes include more knowledge content (e.g., rules and techniques) and less motor content. Also, elementary school students may need more in-class PA promotion in the form of frequent verbal cues and praises/encouragements than high school students. Keeping these factors in mind, we used different cut-points across school levels to define higher motor content and higher in-class PA promotion and found that almost all PE classes (90%) across the school levels with higher motor content and higher in-class PA promotion met the recommended level of MVPA. These findings will help in developing PE guidelines tailored for specific populations.

To further help develop these tailored intervention strategies, the current study revealed a few important details. Consistent with previous studies [13, 14], we found higher MVPA for outdoor lessons in elementary and middle schools. However, it was not the case in high schools. The null association in high schools could be mainly because high schoolers were active even in indoor lessons. Considering that it is often true that high schools have large indoor PE facilities, specific elements of the indoor space could have affected the findings. In this study sample, almost a third of PE lesson time in elementary and middle schools was devoted to general content. The time spent in transitional and management content can be minimized by improving teachers’ class management skills, so that more time can be spent in motor content. In terms of specific motor content, the current study suggests that more fitness content will help to increase MVPA in all school levels, while game content will do so only in elementary schools.

Our findings on middle schools is worth attention. Middle school is a transitional period from elementary school to high school. The PE classes of middle schools are as long in length and as large in class size as in high schools. However, middle school students are not as developmentally mature or skillful as high school students. In addition, substantial adolescent changes, which are associated with PA reduction [23], unfold in the middle school years [24]. In the current study sample, middle school PE classes had the lowest MVPA levels. We further found that despite longer PE lessons in middle schools, the number of minutes spent in MVPA was the same as for elementary PE classes. MVPA levels were particularly lower in the lower middle school grades. The change in PE lessons in terms of lesson length and class size may require significant adjustments by students. Also, despite almost twice more time spent in game play in middle schools than elementary schools, game play seems not to contribute to increasing MVPA. Further research should follow to better understand student PA behavior during PE in the transition to middle school.

### Strengths and limitations

This study is one of the largest studies with observation of over 2000 PE classes over time to evaluate the association between environmental characteristics of PE lessons and student PA levels in underserved communities. However, several limitations should be acknowledged. First, student PA levels were measured through SOFIT observations, which could have been subjective. The SOFIT could also have introduced potential error in the evaluation of student activity levels from focal child observation [25]. However, the SOFIT is the most widely used tool to evaluate PA during PE lessons and has been nationally recognized as an appropriate surveillance tool [19]. Second, despite our efforts to achieve consistent SOFIT coding across observers as well as over time within an observer, we failed to record any data to quantify reliability, which could have compromised the quality of the SOFIT data. Third, although we observed over 200 PE lessons in middle schools, these were only based on six schools. Therefore, caution is needed to interpret the middle school results. Fourth, teacher’s participation in the PE teacher training could have affected PE class characteristics and student PA levels. Fifth, despite our efforts to follow the guidelines for SOFIT reporting [11], we did not have data for other environmental conditions (e.g., size of instructional space, available equipment and supplies), teacher characteristics, or the quality of the lesson content, which could have affected student MVPA. Thus, unmeasured confounding could have biased our results. Lastly, the findings from this study may not be generalizable to any other settings or populations.

## Conclusions

PE lessons with higher motor content and higher teacher’s in-class PA promotion are suggested to meet the recommended level of MVPA during PE classes in all school levels. However, some factors were significant only for certain school levels. Outdoor lesson was associated with MVPA ≥50% in elementary and middle schools. Higher game play content was associated with MVPA ≥50% only in elementary schools.

## Supplementary information

**Additional file 1 Table S1** Comparison of the class characteristics for four MVPA levels during PE class

## Data Availability

Not applicable.

## Abbreviations

CI: Confidence interval
MVPA: Moderate- and vigorous-intensity physical activity
PA: Physical activity
PE: Physical education
SOFIT: System for Observing Fitness Instruction Time
