# Supplementary material for: Physical education environment and student physical activity levels in low-income communities
Source: BMC Public Health. 2020 Jan 31;20:147. doi: 10.1186/s12889-020-8278-8 (PMC6995192; doi:10.1186/s12889-020-8278-8)
Supplement: Supplementary file 1 — Additional file 1 Table S1 Comparison of the class characteristics for four MVPA levels during PE class [file 12889_2020_8278_MOESM1_ESM.docx]

Supplementary Table. Comparison of the class characteristics for four MVPA levels during PE class

|  | Elementary school | | | | Middle school | | | | High school | | | |
| --- | --- | --- | --- | --- | --- | --- | --- | --- | --- | --- | --- | --- |
|  | MVPA <25% | MVPA  25-<50% | MVPA  50-<75% | MVPA  ≥ 75% | MVPA <25% | MVPA  25-<50% | MVPA  50-<75% | MVPA  ≥ 75% | MVPA <25% | MVPA  25-<50% | MVPA  50-<75% | MVPA  ≥ 75% |
|  | n (%) | n (%) | n (%) | n (%) | n (%) | n (%) | n (%) | n (%) | n (%) | n (%) | n (%) | n (%) |
| PE class | 129 (9) | 663 (48) | 539 (39) | 55 (4) | 43 (17) | 122 (48) | 78 (30) | 12 (5) | 28 (7) | 138 (33) | 199 (47) | 57 (13) |
| Grade* |  |  |  |  |  |  |  |  |  |  |  |  |
| Lowest | 34 (8) | 188 (46) | 175 (42) | 15 (4) | 10 (15) | 37 (57) | 13 (20) | 5 (8) | NA | NA | NA | NA |
| Middle | 52 (10) | 266 (50) | 188 (36) | 20 (4) | 19 (17) | 56 (50) | 32 (29) | 4 (4) | NA | NA | NA | NA |
| Highest | 43 (10) | 209 (47) | 176 (39) | 20 (4) | 14 (18) | 29 (36) | 33 (42) | 3 (4) | NA | NA | NA | NA |
| Lesson location |  |  |  |  |  |  |  |  |  |  |  |  |
| Outdoor | 2 (1) | 40 (24) | 101 (61) | 22 (13) | 2 (4) | 16 (33) | 26 (55) | 4 (8) | 1 (3) | 14 (38) | 20 (54) | 2 (5) |
| Indoor | 127 (10) | 623 (51) | 438 (36) | 33 (3) | 41 (20) | 106 (51) | 52 (25) | 8 (4) | 27 (7) | 124 (32) | 179 (46) | 55 (14) |
|  | M±SD | M±SD | M±SD | M±SD | M±SD | M±SD | M±SD | M±SD | M±SD | M±SD | M±SD | M±SD |
| Male students observed, n | 2±1 | 2±1 | 2±1 | 2±1 | 2±1 | 2±1 | 2±1 | 2±1 | 2±1 | 2±1 | 2±1 | 2±1 |
| Class size (# students), n | 22±8 | 22±8 | 22±7 | 21±8 | 31±15 | 45±27 | 53±31 | 65±32 | 35±9 | 35±16 | 38±20 | 32±25 |
| Students per teacher, n | 20±6 | 20±6 | 21±6 | 20±8 | 26±9 | 35±19 | 36±24 | 27±4 | 32±7 | 32±13 | 32±15 | 25±15 |
| Actual length of PE class, min | 30±5 | 31±6 | 32±5 | 32±4 | 36±5 | 42±15 | 44±20 | 32±11 | 39±11 | 41±12 | 43±14 | 34±8 |
| In-class PA promotion, % | 7±10 | 18±18 | 23±21 | 24±24 | 5±5 | 7±11 | 25±23 | 34±16 | 1±2 | 5±8 | 12±15 | 16±15 |
| General content, % | 41±18 | 35±14 | 24±10 | 15±7 | 44±20 | 35±13 | 24±10 | 15±6 | 39±24 | 27±12 | 22±10 | 17±9 |
| Knowledge content, % | 16±12 | 17±11 | 11±9 | 5±4 | 3±6 | 6±10 | 9±7 | 5±3 | 4±8 | 3±6 | 3±5 | 1±2 |
| Motor content, % | 43±19 | 49±15 | 64±13 | 80±8 | 53±22 | 60±14 | 68±12 | 80±6 | 58±24 | 70±14 | 75±12 | 82±10 |
| Fitness, % | 15±19 | 20±19 | 32±20 | 51±22 | 15±13 | 15±12 | 34±20 | 53±27 | 24±27 | 24±24 | 30±26 | 40±35 |
| Skill practice, % | 10±15 | 12±17 | 9±15 | 8±18 | 3±9 | 13±20 | 13±20 | 6±14 | 3±11 | 5±15 | 3±12 | 1±11 |
| Game play, % | 10±18 | 12±17 | 19±19 | 13±16 | 33±30 | 34±27 | 15±19 | 21±27 | 18±30 | 31±29 | 32±28 | 27±35 |
| Other, % | 8±18 | 4±14 | 5±17 | 8±15 | 2±8 | 3±12 | 5±17 | 0±0 | 12±23 | 10±23 | 11±24 | 14±30 |

M±SD; mean±standard deviation; MVPA, moderate- and vigorous-intensity physical activity; NA, not applicable; PA, physical activity; PE, physical education.

*For elementary school, the lowest was grades K-1, the middle was grades 2-3 and the highest was ≥grades 4-5. For middle schools, the lowest was grade 6, the middle was grade 7, and the highest was grade 8. Because most high school PE lessons were grade-mixed, MVPA level by grade was not analyzed for high schools.
